# Utilisation of Oxford Nanopore sequencing to generate six complete gastropod mitochondrial genomes as part of a biodiversity curriculum

**DOI:** 10.1038/s41598-022-14121-0

**Published:** 2022-06-15

**Authors:** Mattia De Vivo, Hsin-Han Lee, Yu-Sin Huang, Niklas Dreyer, Chia-Ling Fong, Felipe Monteiro Gomes de Mattos, Dharmesh Jain, Yung-Hui Victoria Wen, John Karichu Mwihaki, Tzi-Yuan Wang, Ryuji J. Machida, John Wang, Benny K. K. Chan, Isheng Jason Tsai

**Affiliations:** 1 Biodiversity Research Center, Academia Sinica, Taipei, Taiwan; 2 Department of Life Science, National Taiwan Normal University, Taipei, Taiwan; 3 Biodiversity Program, Taiwan International Graduate Program, Academia Sinica and National Taiwan Normal University, Taipei, Taiwan; 4 Bioinformatics Program, Taiwan International Graduate Program, National Taiwan University, Taipei, Taiwan; 5 Bioinformatics Program, Institute of Information Science, Taiwan International Graduate Program, Academia Sinica, Taipei, Taiwan; 6 Natural History Museum of Denmark, University of Copenhagen, Faculty of Science, Copenhagen, Denmark; 7 Institute of Plant and Microbial Biology, Academia Sinica, Taipei, Taiwan; 8 Graduate Institute of Biotechnology, National Chung-Hsing University, Taichung, Taiwan; 9 Molecular and Biological Agricultural Sciences, Taiwan International Graduate Program, Academia Sinica and National Chung Hsing University, Taipei, Taiwan; 10 Ph.D. Program in Microbial Genomics, National Chung Hsing University and Academia Sinica, Taipei, Taiwan

**Keywords:** Molecular ecology, Genomics, Biodiversity, Sequencing, Evolutionary genetics, Taxonomy

## Abstract

High-throughput sequencing has enabled genome skimming approaches to produce complete mitochondrial genomes (mitogenomes) for species identification and phylogenomics purposes. In particular, the portable sequencing device from Oxford Nanopore Technologies (ONT) has the potential to facilitate hands-on training from sampling to sequencing and interpretation of mitogenomes. In this study, we present the results from sampling and sequencing of six gastropod mitogenomes (*Aplysia argus, Cellana orientalis, Cellana toreuma, Conus ebraeus, Conus miles* and *Tylothais aculeata*) from a graduate level biodiversity course. The students were able to produce mitogenomes from sampling to annotation using existing protocols and programs. Approximately 4 Gb of sequence was produced from 16 Flongle and one MinION flow cells, averaging 235 Mb and N50 = 4.4 kb per flow cell. Five of the six 14.1–18 kb mitogenomes were circlised containing all 13 core protein coding genes. Additional Illumina sequencing revealed that the ONT assemblies spanned over highly AT rich sequences in the control region that were otherwise missing in Illumina-assembled mitogenomes, but still contained a base error of one every 70.8–346.7 bp under the fast mode basecalling with the majority occurring at homopolymer regions. Our findings suggest that the portable MinION device can be used to rapidly produce low-cost mitogenomes onsite and tailored to genomics-based training in biodiversity research.

## Introduction

Species identification is a key process across biological disciplines^1–5^. Currently, species identity is confirmed through combining morphological and molecular information. In animals, the latter most concerns using mitochondrial markers^6^, given their presence in high quantities in metazoan cells, elevated rates of molecular evolution^6,7^, lack of recombination and ease to sequence compared to nuclear markers^1,7–9^. The nuclear ribosomal ITS region can be also used as a marker, although its short length and fast evolution limits comparisons to the species and genus levels^1,8^ Driven by rapidly improving sequencing technologies and decreasing per-base sequencing costs, an approach in which a genome is sequenced to low coverage (often ~ 1 ×) called genome skimming is now available for retrieving and assembling complete mitogenomes from animal samples^10,11^. Other strategies for obtaining mitogenomes include the use of PCR of mitochondrial amplicons followed by Sanger or Illumina sequencing or shotgun Illumina sequencing, all of which can be time-consuming and expensive. In particular, PCR strategies require lab reagents and specific primers which may not be present in all the laboratories^10,12,13^ and it can be also hard to teach^14^, while also being prone to errors^15^.

Third-generation sequencing from Oxford Nanopore Technologies (ONT), which allows for long reads to be generated with simple setup, is particularly suitable for sequencing mitogenomes at lower cost by genome skimming^12,17^. It is also more rapid compared to other methods^12,16,17^. For species identification, ONT has been successfully used for amplifying mitogenomes for vertebrates^12^ and arthropods^13,18^. Portability has been achieved by ONT with its MinION device, making this technology especially attractive for teaching DNA sequencing and assembly virtually anywhere^19–21^. Additionally, MinION is cheaper compared to other sequencing methods (i.e., Illumina), which may require the service of a company outside the lab^22^. For example, the Flongle flow cell costs less than 100 USD per flow cell and allows the generation of up to 2.8 Gb of data^23^, while the MinION flow cell costs around 1000 USD, which is generally cheaper than Illumina MiSeq sequencing services^22^, and can generate up to 50 Gb of data^24^. A previous limitation of ONT sequencing compared to other systems was a high raw sequencing error rate, ranging from 5 to 15% compared to 0.3% for Illumina^25^. This is constantly reducing which can be further corrected with Illumina short reads^12^ or by polishing with increased DNA coverage^12,13,25^. While some mitogenomes have been assembled through ONT reads only^12,13^, the general consensus has been to combine both short and long reads in a hybrid approach^25^.

Despite the potential to address genome deficiencies in non-model organisms and for comprehensive species delimitation, ONT mitogenome sequencing is yet to be tested across clades in which it would be extremely beneficial. A taxon of particular interest is the phylum Mollusca^26^. It is the second species-rich animal phylum, with around 117,000 described species and an estimated 150,000 undescribed marine ones^27,28^ and has critical ecological, cultural and economic importance^29–35^. According to GenBank (^36^, last assessed 27th January 2022), there are 845 mitogenomes (sequences from 13,000 bp onward) available for Gastropoda, 604 for Bivalvia, 224 for Cephalopoda, 4 for Scaphopoda, 3 for Monoplacophora, 24 for Polyplacophora and 9 for Aplacophora. These data have played an important role in understanding evolution in molluscan sub-classes^26,37^. Yet, due to considerable size variation, notable rearrangements, gene duplications and losses as well as reported cases of doubly uniparental inheritance in bivalves, molluscs harbor some of the most complex mitogenomes among metazoans^26^. Given these features, the long reads generated by ONT sequencing (sometimes getting the whole mitochondrial sequences from a single long read^15^) should be useful for fixing annotation mistakes, as done in other groups^38^, and help to understand the extent of tandem duplications^26^.

Here, we establish a system for ONT sequencing of gastropod mitogenomics useful for rapid species identification and mitogenome characterization in a teaching context. We developed a graduate-level curriculum class to specifically address challenges associated with ONT sequencing and assembly and report six high-quality mitogenomes of diverse members of Gastropoda. To assess the accuracy of these ONT assemblies, we produced additional Illumina sequences and compared the extent and nature of sequencing errors and their impacts on mis-assemblies.

## Results

### Sampling and morphological identification of six gastropods

In March 2021, eight graduate students took a sampling trip to Dai Bai Sha on Green Island, Taiwan (Supplementary Fig. S1). Five gastropod species belonging to four families within Gastropoda were collected and morphologically identified (Table 1). The students extracted genomic DNA and sequenced it using Flongle flow cells. We noted that prior to the class a sample DJ was collected in Ruifang, Taiwan to test the whole procedure, resulting in a total of six species presented in this study. A more formal description of the sampling trip and morphological descriptions are described in Supplementary Info.

**Table 1:** Sample identification (ID) codes, together with original morphological identification and BLASTn results of the *cox1* sequence.

Sample ID	Aoc	Cra	DJ	Ceb	Cfl	Mku
Family	Aplysidae	Nacellidae	Nacellidae	Conidae	Conidae	Muricidae
Initial morphological identification	*Aplysia oculifera*	*Cellana radiata*	*Cellana toreuma*	*Conus ebraeus*	*Conus flavidus*	*Mancinella* sp.
**Uncorrected ONT**						
*cox1* top hit*	*Aplysia argus*	*Cellana radiata orientalis*	*Cellana toreuma*	Conus ebraeus**	*Conus miles*	*Thais aculeata*
Bit Score (fast)	1175	1186	1042	1151	1158	1136
Nucleotide identity (%) (fast)	98.9	99.1	99.3	99.2	99.5	98.0
Bit Score (hac)	1194	1199	1066	1151	1170	1197
Nucleotide identity (%) (hac)	99.4	99.5	100	99.4	99.8	99.5
**Final assembly**						
Genbank Accession	ON018801	ON018804	ON018805	ON018802	ON018803	ON018806
Length (bp)	14,124	16,169	16,268	18,031	16,243	17,024
AT content (%)	66.5	69.5	68.4	67.5	61.8	67.0
Bit Score	1194	1205	1240	1175	1170	1205
Nucleotide identity (%)	99.4	99.7	100	100.0	99.8	99.7

*Latest species names are provided in the table; some were not yet updated in GenBank. **Conus cloveri with 87.2% nucleotide identity was identified as top hit when the full *cox1* sequence was used. We searched instead using Folmer region and identified *C. ebraeus* with much higher nucleotide identity.

### Five out of six circular mitogenomes of gastropods

During the sampling trip, the students initially conducted on-site ONT sequencing of one Flongle flow cell per species which resulted in 30.4–315.3 Mb of sequences per species (Supplementary Table S1). Additional sequencing of 2–4 flow cells used per species was obtained until a full (or nearly full) assembly was produced. A total of 16 Flongle and one MinIon flow cells were used, yielding an average of 235 Mb of sequence basecalled with fast mode with an average read length N50 of 4.4 kb. Variation in sequencing yield and sequence length differences were observed between species and flow cells (Supplementary Table S2). After filtering for putative mitochondrial reads using the mitogenomes of the most closely related species available in the NCBI database using DIAMOND^39^, approximately 11–47 × depth of coverage was obtained for each species corresponding to 0.02–4.8% of on-target sequencing (Supplementary Table S2). Assembly using Flye^40^ produced circlised mitogenomes in five out of the six species (Supplementary Table S3), confirming that sequencing mitogenomes were achievable in a classroom setting using only sequences from Flongle flow cells and two published programs. Annotations using MitoZ and MITOS^41,42^ revealed that five sequences were complete with the presence of 13 protein-coding genes, 22 tRNAs and two rRNAs. An exception was the Cfl sample, which had an incomplete mitogenome lacking the *nad5*, tRNA^His^ and tRNA^Phe^ genes (Supplementary Table S3). At the end of the bioinformatics exercise, students took the annotated *cox1* nucleotide sequences and identified the most similar sequences available in the NCBI database via BLASTn or BLASTp. Partial *cox1* sequences with 98–99.3% nucleotide identity were obtained in these six samples, providing additional information for species identification (Table 1). Three samples (Aoc, Cfl and Mku) had results conflicting with the original morphological identification, which required additional information or phylogenetic analyses to resolve these issues.

Inspection of the annotations from ONT-only assemblies revealed the presence of extensive premature stop codons in every annotated protein-coding gene. As a result, only 12–40% of *cox1* query coverage matched a *cox1* homolog in the NCBI nr database using BLASTp (Supplementary Table S4). Using the high accuracy (hac) mode, the number of basecalled sequences were on average 13–35% less than from fast mode corresponding to 7–43 × depth of coverage in each species. Assemblies of the hac-mode base-called sequences using the same pipeline produced four circlised mitogenomes (out of six), and annotations contained mis-assemblies such as duplicated or truncated genes in five species (Supplementary Table S5). However, overall nucleotide identity to *cox1* sequence matches increased to 99.4–100% (Table 1). The only assembly without mis-assembly was sample Aoc with the highest mitogenome sequencing coverage of 43 ×, suggesting mis-assembly was caused by insufficient coverage in the rest of the five samples.

### Quantifying the extent of nanopore errors

**Figure 1:**
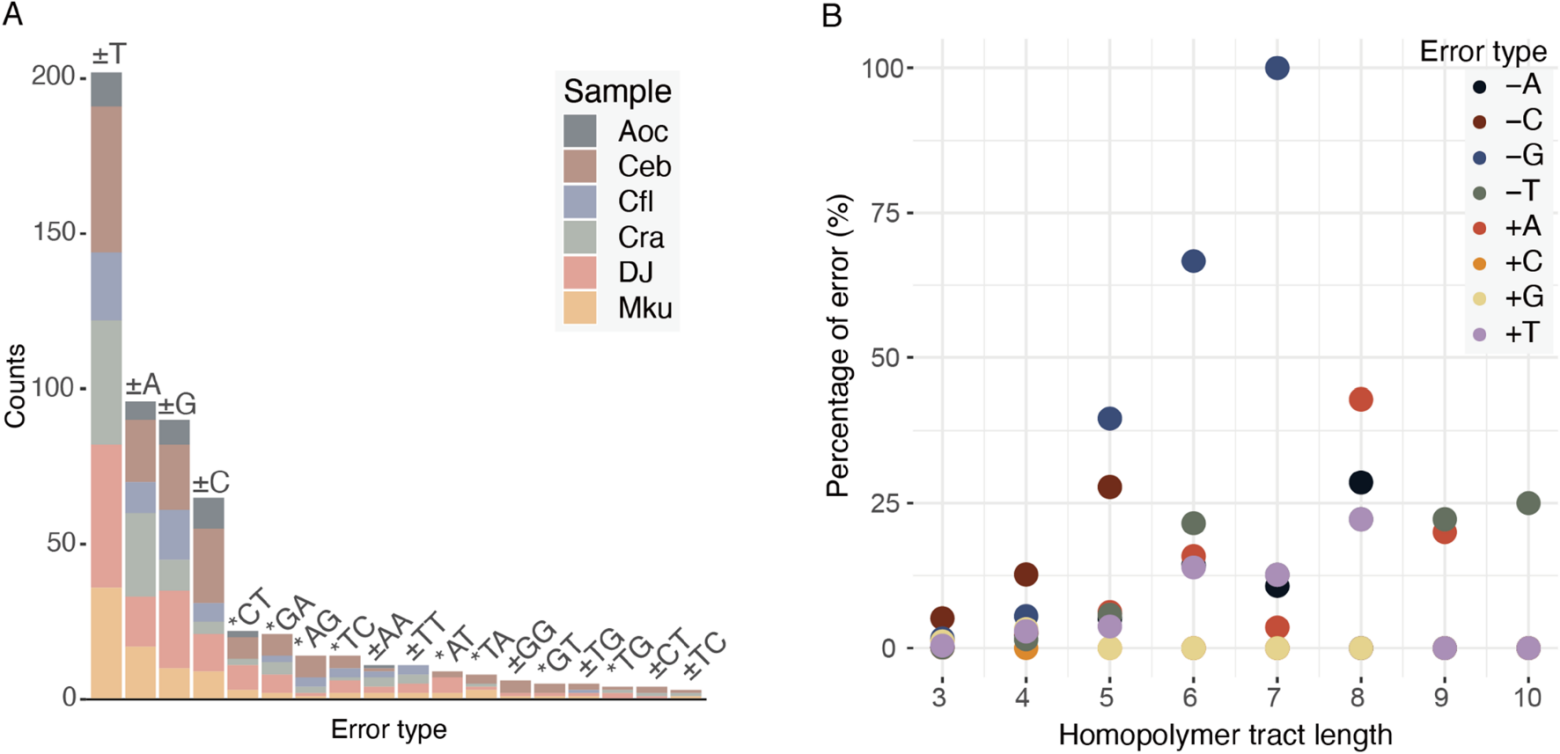
Quantification of ONT errors from fast mode basecalling. (**A**) Number of INDELs (+/−) and substitutions (*) in ONT assemblies before and after consensus improvement using Illumina reads. Error types that occurred once (n = 15) and twice (n = 8) were excluded from the plot. (**B**) Relationship between composition of single-base INDELs and homopolymer length.

To quantify and correct the extent of errors, we further sequenced the six Gastropoda samples using the Illumina platform. A total of 52.8–973.2 × depth of mitochondrial reads were obtained (Supplementary Table S2), which were used to de novo assemble mitogenomes from Illumina data only as well as to polish the ONT-only assemblies. The consensus quality values (QVs) of Nanopore assemblies in fast and hac modes were 18.5–25.4 and 25.7–38.0, which corresponded to one base error every 70.8–346.7 and 371.5–6309.6 bp, respectively. Polishing of the ONT-only assemblies from the fast basecalling mode using the Illumina sequences resulted in 41–226 modified sites in each species. Comparison of the original to the polished ONT assemblies revealed that the errors were non-random, with single base indels dominating (66.7–85.4%) (Fig. 1A). Of these, single T and A indels comprised 48% of the total errors presumably because of the high AT composition of mitogenomes (Table 1). The majority (71.2%) of errors were located at homopolymer regions (Supplementary Fig. S2), consistent with previous observations of mitogenome assemblies using ONT technologies^43^. As expected, we observed a positive trend of errors being called with increasing homopolymer length (Fig. 1B) suggesting it was challenging to basecall precisely in these regions. Despite the mis-assemblies, only 2–44 sites were modified on the ONT assemblies produced from reads basecalled in hac mode with similar error profiles to the fast mode (Supplementary Fig. S3).

**Figure 2:**
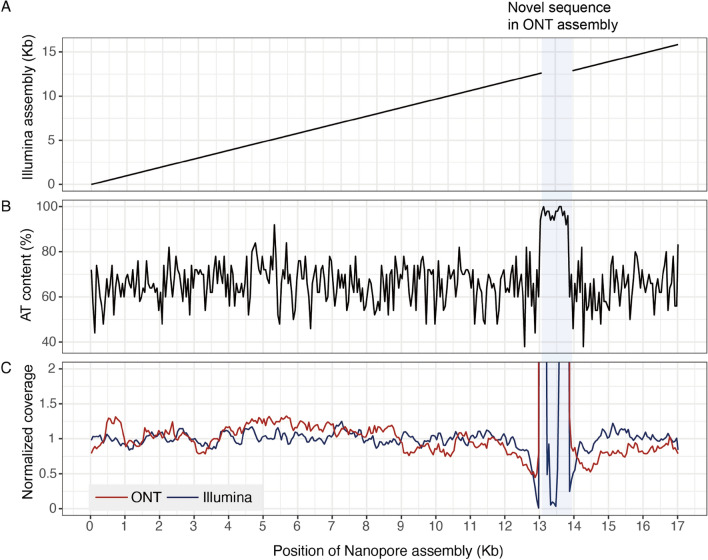
ONT assembly features of sample Mku. (**A**) Dotplot against Illumina assembly. (**B**) AT content in 50 bp windows. (**C**) Nanopore and Illumina read coverage in 50 bp windows.

We sought to access assembly completeness by comparing the assemblies produced solely from Illumina reads and the polished ONT assemblies derived from fast mode as they contained less mis-assemblies. In three samples (Aoc, Cra, and DJ), consistent sequences (nucleotide identity 99.9–100% covering 99.6–100% of sequence) were observed with both technologies, indicating the assemblies made on these sequences were robust. However, in the Ceb and Mku samples, additional sequences of length 2169 bp and 868 bp, respectively were found present only in the ONT assembly (Fig. 2A and Supplementary Fig. S4). The additional sequences are highly AT rich (98.7%; Fig. 2B) and harbors low Illumina read coverage (Fig. 2C), consistent with the known property that this technology has difficulties sequencing over regions with highly biased base composition^43^. Despite ONT technology being able to sequence over these regions, a mis-assembly was observed in another sample, Cfl, where one core gene was missing and three were duplicated (Supplementary Table S3). In contrast, the Cfl assembly produced from Illumina reads resulted in all core genes annotated as single copies. The mis-assembly was likely because Cfl had the lowest ONT sequencing N50 (1.2 kb) of all the samples despite 27.5 × depth of coverage (Supplementary Table S2). In comparison, sample DJ produced a circlised assembly with the longest ONT N50 of 8.3 kb despite having the lowest depth of mitogenome coverage (11 ×) amongst samples. For the remainder of the analyses, annotations from polished Nanopore assemblies will be used with the exception of sample Cfl (Table 1 and Supplementary Table S6). BLASTn results of the polished *cox1* sequences showed an increase of 0.3–1.7% nucleotide identity to the same top matched sequences in the uncorrected ONT assemblies (Table 1), presumably because the erroneous bases were corrected. As expected, query coverage of the top *cox1* hits in BLASTp improved considerably to 99–100% in the final assemblies since they contained no premature stop codons (Supplementary Table S4). Together, these results suggest that, currently, a hybrid sequencing approach should be still employed in order to obtain an accurate and complete mitogenome.

### Phylogenomics of gastropod mitogenomes

**Figure 3:**
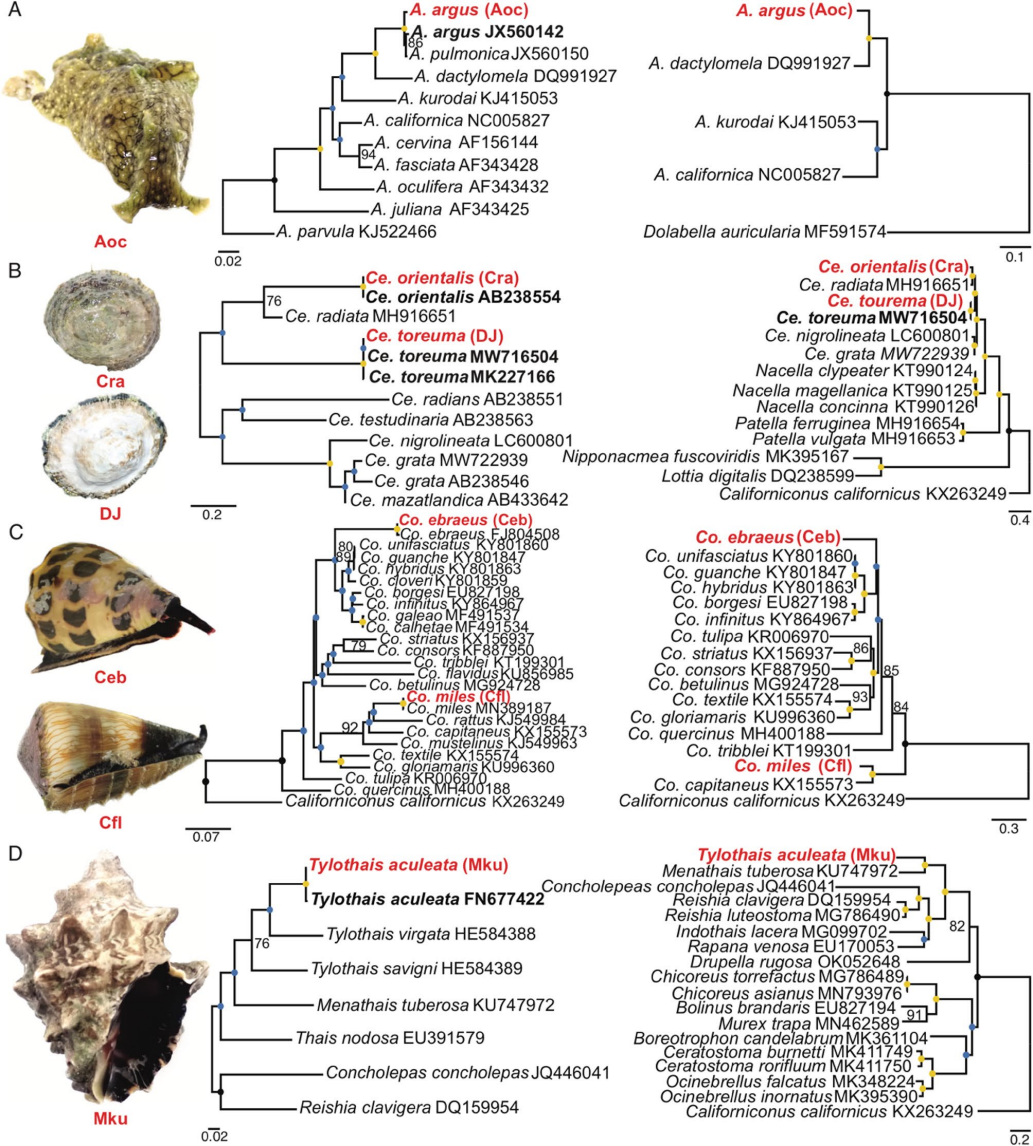
*cox1* (left) and mitogenome (right) phylogenies from each family. From top to bottom: (**A**) Aplysiidae (with *Aplysia argus*); (**B**) Patellogastropoda (with *Cellana orientalis* and *Cellana toreuma*); (**C**) Conidae (with *Conus ebraeus* and *Conus miles*); and (**D**) Muricidae (with *Tylothais aculeata*). Blue dots represent bootstrap support ≤ 75, yellow ones represent bootstrap support ≥ 95. Values in the middle are written. Red bold tips represent our specimens, black bold ones represent the identified species’ sequences.

To better resolve species relationships in each family, we constructed a maximum likelihood *cox1* phylogeny using nucleotide alignments and mitogenome phylogenies either using concatenated codon alignments of all protein coding genes or coalescence of individual gene phylogenies of representative species (Supplementary Table S7). In general, congruence was observed between the *cox1* and mitogenome phylogenies, with higher bootstrap support values in the latter (defined here as more nodes with bootstrap > 75; Fig. 3; Supplementary Figs. S5–S8). With the exception of the DJ sample, all the assemblies reported in this study were the first complete mitogenomes for the designated species.

Examination of the phylogenetic trees revealed additional information behind four incongruences between the initial morphological suggestion and the *cox1* top hits. The first was our *Aplysia* species (Aoc, a sea hare), which was originally identified as *A. oculifera* based on the presence of the ring spots alone (Table 1 and Fig. 3A, Supplementary Info). We redesignated this sample as *A. argus* (Fig. 3A) which is the current name used to distinguish the individuals previously recognised as *A. dactylomela* and *A. pulmonifera*’s Indo-Pacific specimens^44^, consistent with the clustering in the phylogenies. Second, sample Cra was redesignated as *Cellana orientalis* (Fig. 3B), which was once regarded as a subspecies of *Ce. radiata* but is now described as an independent species^45^. Third, one of the *Conus* specimens Cfl was initially identified as *Conus flavidus* and redesignated as *Co. miles* (Fig. 3C). Finally, the murex snail (sample Mku) was tentatively recognised as a species belonging to the genus *Mancinella* in the taxonomically challenging family Muricidae^46^. We redesignated this sample as *Tylothais aculeata* (Fig. 3D, Supplementary Info) which was recently erected from *Thalessa*^47^ and previously regarded as a *Mancinella* species in Taiwan^48^. The Muricidae mitogenome phylogeny was consistent with previous classification, clustering species in the subfamily Rapaninae, Ocenebrinae and Muricinae (Fig. 3D^46^).

### Synteny of mitogenomes

**Figure 4:**
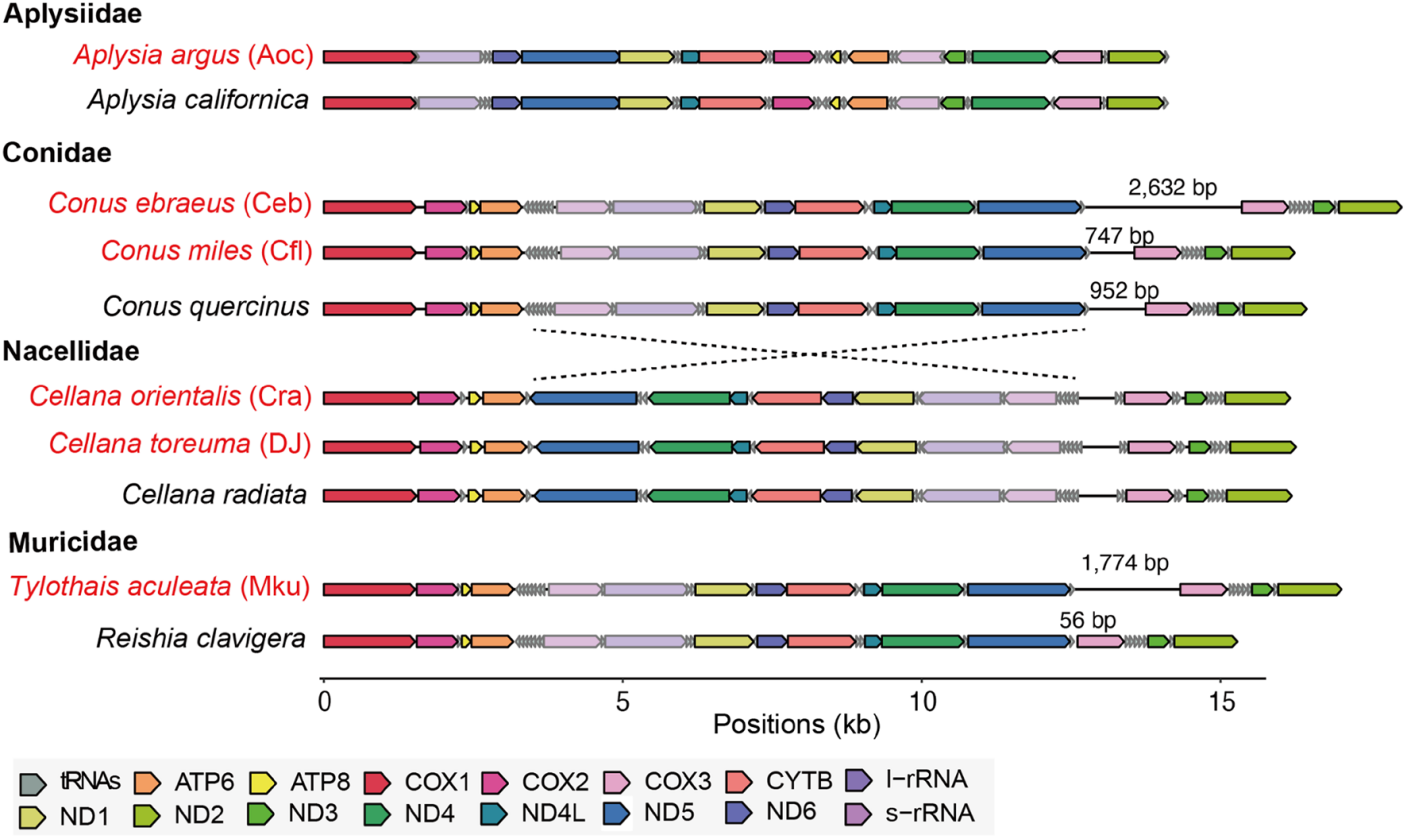
Synteny comparison among our samples and reference mitogenomes. Red labels denote our samples. The lengths of the control region between tRNA^Phe^ and *cox3* are shown when more than 1kb difference are observed between closely related species.

The availability of complete mitogenomes allowed us to assess their synteny with sister species and between families. We inspected synteny amongst complete mitogenomes of three Patellogastropoda families (Nacellidae, Patellidae, and Lottidae) and found a general consistency with those from previous studies (Fig. 4 and Supplementary Fig. S9^49–51^). For example, the most apparent difference, the highly rearranged mitogenomes in Lottidae compared to other Patellogastropoda families, with one large inversion of all protein-coding genes (except *cox1* and *cox3*) between *Nipponacmea fuscoviridis* and *Lottia digitalis* (Supplementary Fig. S9), was already acknowledged^51^. Interestingly, the control region between tRNA^Phe^ and *cox3*  typically observed in Gastropoda mitogenomes were much longer in two of our ONT assemblies with the aforementioned novel AT-rich sequences (Figs. 2 and 4), suggesting hidden diversity present in this region that were previously nearly invisible to Illumina technologies.

## Discussion

The primary purpose of this study was to assess whether ONT can be used in a biodiversity curriculum as a reliable tool for generating accurate mitogenomes for expanding resources for the research community. Although multiple assemblies can be constructed and merged in order to achieve greater consensus quality^52,53^, we show that closed (i.e., circular) mitogenomes can be achieved with a simple preconstructed bioinformatic pipeline for teaching purposes. This allowed the students to undertake the pipeline and complete the tasks within a typical lecture of three hours. The subsequent polished assemblies can serve as new accurate resources for the research community. Hence, this work highlights that incorporating ONT sequencing in genome skimming approaches holds great potential for exploring and populating sequence databases with the mitogenomes while integrated with educational purposes.

As this was our first attempt to combine ONT with field sampling, sequencing and teaching, we did not target specific taxa and opted for the field sequencing kits that are designed for simple operation and time-efficiency but may compromise the quality of extracted genomic DNA. With students having no *a priori* experience, variation in sequencing yields were anticipated. Despite the prevalence of single base errors under the fast-basecalling mode, ONT has one advantage over Illumina technology, which is that the sequencing of highly AT-rich sequences is not biased. The novel AT-rich sequences in *C. ebraeus* and *T. aculeata* coincided with the control region amongst published assemblies (Fig. 4) and implies a re-assessment using different sequencing technologies may be useful. Recently, long read sequencing has corrected errors in at least 100 reference mitogenomes^38^. Given the anticipated increased performance as ONT matures, confirmation and validation with additional ONT sequencing may be built into part of the teaching curriculum to specifically address samples that have suspect control region assemblies.

Several improvements in the quality of ONT-only mitogenome assembly can be made beyond the teaching context. A mitogenome consensus with overall better accuracy was produced through multiple passes and comparisons of assemblies using different programs^12^, although some level of manual inspections was required. Given sufficient sequencing coverage, we found basecalling with high accuracy (hac) mode was able to produce mitogenomes with complete genes without premature stop codons. In addition to enriching for mitochondrial DNA experimentally^15,54^, the use of adaptive sampling recently developed in ONT allows preselected sequences to be enriched during the sequencing process^55–57^. This approach has proven successful in obtaining full mitogenomes of endangered animals by enriching host genomic DNA from fecal samples^56^. Bait sequences can be up to 30% sequence identity divergent, suggesting the feasibility of this approach for sequencing an unknown species using references from distant relatives^55^. Improvement in read accuracy, in particular at homopolymeric regions, was observed in the recent new ONT R10.4 sequencing chemistry allowing near perfect bacterial assemblies^58^. Nevertheless, we recommend additional Illumina sequencing should be still employed if incomplete genes were identified.

In conclusion, this study shows that ONT can also be a tool for students to learn how to work with and sequence DNA directly in a field station, thus making it fit as part of a graduate-level class and curriculum in biology and bioinformatics. With continuous improvement in read accuracy and yield in long read technologies, we anticipate one day that new accurate and complete mitogenomes may rapidly populate the Tree of Life across different corners of the world by users ranging from evolutionary biologists to citizen scientists to high school students.

## Material and methods

### Sampling processing, DNA extraction and sequencing

Sampling of six gastropods by the students is detailed in Supplementary Info. The solutions used to extract high-yield genomic DNA for mitogenome sequencing were prepared prior to field sampling and DNA extraction following the manufacturer's instructions. We used the Quick-DNA™ HMW MagBead Kit (Catalog No. D6060) for DNA extraction and then the DNA samples were stored in a fridge (4ºC) before Nanopore mitogenome sequencing.

For Nanopore long read sequencing, ~ 400 ng of genomic DNA per sample were used for library construction. Sequencing library was generated using the Field Sequencing kit (SQK-LRK001, Oxford Nanopore Technologies, UK), following the manufacturer's instructions. 30 μl or 75 μl of the library were loaded into a Flongle (FLO-FLG001) or partially used MinION (FLO-MIN106 with 356 pores left) flow cells, respectively. Each library was sequenced by a MinION device for 24–48 h. The ONT FAST5 output files were converted to FASTQ files using Guppy 4.4.2^59^ in fast and hac mode with default setting (Oxford Nanopore Technologies, Oxford, UK). Both DNA extraction and the initial sequencing were done in the Green Island Marine Research Station, Marine Science Center, Academia Sinica, Taiwan. Additional ONT sequencing was done on the main Academia Sinica campus.

For Illumina short reads sequencing, ~ 200 ng DNA per sample was used for the DNA library preparations. Sequencing libraries were generated using TruSeq Nano DNA HT Sample Prep Kit (Illumina USA) following manufacturer’s recommendations and index codes were added to each sample. Briefly, genomic DNA sample was fragmented by sonication to 350 bp. Then DNA fragments were end-polished, size selected, A-tailed, and ligated with the full-length adapter for Illumina sequencing, followed by further PCR amplification. After PCR products were purified (SPRIselect reagent, Beckman), libraries were analysed for size distribution by Agilent 2100 Bioanalyzer and quantified by Qubit. The DNA libraries were sequenced on the Illumina NovaSeq 6000 platform and 150 bp paired end reads were generated by Genomics BioSci & Tech Co. Illumina reads were trimmed by fastp (ver. 0.22^60^) with default parameters.

### Assembly and annotation of gastropod mitogenomes

Amino acid sequences of the complete mitogenomes of sister species to the samples were obtained from NCBI (Sample Aoc: *Aplysia californica* NC005827.1; Ceb and Cfl: *Conus quercinus* NC035007.1; Cra and DJ: *Cellana radiata* MH916651.1; and Mku: *Reishia clavigera* NC010090.1). These sequences served as baits to search for putative mitochondrial sequences using DIAMOND (ver. 0.9.24.125^39^). An initial assembly was produced from these putative mitochondrial sequences using Flye (ver. 2.8.3^40^) and served as baits to search for all possible mitochondrial sequences using Minimap2 (ver. 2.24; options: -x map-ont^61^). A second round of ONT assemblies were produced and further polished using the same set of data by racon (ver. 1.4.11^62^) for four iterations and medaka (ver. 1.2.0^63^). A final round of polishing was conducted using Pilon (ver. 1.22^64^) with Illumina reads. Assemblies using solely Illumina reads were generated using MitoZ (ver. 2.4-alpha^41^). Both versions of assemblies were subjected to MitoZ (options: --clade Mollusca) for annotation. The one which had better sequence integrity and gene completeness was selected as the final version. Gene annotations on final assemblies were further curated manually to ensure correctness. Read mappings for long and short reads were performed using Minimap2 (ver. 2.24; options: -x map-ont^61^) and bwa (ver. 0.7.17^65^), respectively. Duplicates in Illumina mappings were marked by SAMBLASTER (ver. 0.1.26^66^). The estimation of read coverage was conducted by Mosdepth (ver. 0.2.5^67^). The comparison between assemblies was conducted using Minimap2 (options: -x asm5 --cs) and the paf format output was parsed. Part of the pipeline was redesigned as a three-hour lecture available at^68^ and detailed in Supplementary Info.

### Phylogenetic and synteny analysis

We used 13 mitochondrial protein-coding sequences to construct trees within the gastropod families Aplysiidae, Conidae, Muricidae and subclass Patellogastropoda. Mitogenomes within family Aplysiidae (5), Conidae (18) and Muricidae (17) and within subclass Patellogastropoda (13) were selected as references and downloaded from GenBank (^36^; last assessed: 18th February 2022). The details of downloaded references are shown in Supplementary Table S7. Concatenated and coalescence methods were applied to codon alignments of 13 protein encoding genes in our newly sequenced samples and reference sequences. Sequence alignments for each mitochondrial protein-coding gene was performed using the L-INS-i algorithm in MAFFT 7.487^69^. We concatenated the genes by using SequenceMatrix^70^ and then built Maximum Likelihood phylogenies using ModelTest and RAxML-NG implemented in raxmlGUI^71^, with 500 bootstraps replicates. A consensus tree based on coalescencing all individual gene phylogenies were constructed with ASTRAL^72^. The trees were visualised with FigTree 1.4.4^73^. Gene order of mitogenomes were visualised using the gggenomes package^74^.

We downloaded *cox1* sequences from GenBank for checking the species ID. The sequences were chosen according to a BLASTn search^75^ with default settings. The alignment was performed with MAFFT 7.471^69^ and trimmed manually while inspecting the alignments under MEGA X (ver. 10.1.8^76^). In total, 646 bp were used for reconstructing the *Aplysia* *cox1* tree, 636 for Conidae, and 657 for both Muricidae and *Cellana*. After that, we used ModelTest and RAxML-NG implemented in raxmlGUI^71^ for building a Maximum Likelihood phylogeny for each clade, with 500 replicates. If there were issues with scientific names (i.e., synonyms), the ones accepted by the World Register of Marine Species were used^45^.

## Supplementary Information

Supplementary Information.

Supplementary Tables.

## Data Availability

The final mitogenomes generated and analysed during the current study are available in the GenBank repository with accession number ON018801, ON018804, ON018805, ON018802, ON018803 and ON018806.
