# Supplementary material for: Utilisation of Oxford Nanopore sequencing to generate six complete gastropod mitochondrial genomes as part of a biodiversity curriculum
Source: Sci Rep. 2022 Jun 15;12:9973. doi: 10.1038/s41598-022-14121-0 (PMC9200733; doi:10.1038/s41598-022-14121-0)
Supplement: Supplementary file 1 — Supplementary Information. [file 41598_2022_14121_MOESM1_ESM.pdf]

Utilisation of Oxford Nanopore sequencing to generate six complete gastropod mitochondrial genomes as part of a biodiversity curriculum

Mattia De Vivo*, Hsin-Han Lee*, Yu-Sin Huang*, Niklas Dreyer, Chia-ling Fong, Felipe Monteiro Gomes de Mattos, Dharmesh Jain, Yung-Hui Victoria Wen, John Karichu Mwihaki, Tzi-Yuan Wang, Ryuji J. Machida, John Wang, Benny K. K. Chan and Isheng Jason Tsai

*Equal contributions

Corresponding author: Isheng Jason Tsai, ijtsai@sinica.edu.tw

This file includes:
1. Supplementary Text: Extended Results
2. Supplementary Figure S1 to S9

Other supplementary materials for this manuscript includes:

Supplementary Tables are in a merged Excel file.

Supplementary Text

Field sampling

Prior to the start of a graduate class taught at Taiwan International Graduate Program for Biodiversity [1], one sample (DJ) was collected and sequenced in Ruifang (25°07'17.8"N, 121°49'19.9"E), Taiwan to test the protocols laid out in this study. All materials were used for this sample, hence no voucher was kept. The students went on and collected five Gastropoda samples from the rocky shores in Da Bai Sha (Green Island, Taiwan, 22.639° N, 121.493° S; WGS84, see Supplementary Figure S1) during low tide on 22nd March 2021. Specimens were either collected by hand or with tweezers. The specimens were placed in 2-5 L transparent bottles with sea water and transported back to the lab at the Green Island Marine Research Station, Marine Science Center, Academia Sinica, Taiwan. Here, they were separated by taxon and kept in 10-20 L aquaria in aerated seawater. The specimens were not fed prior post-sampling processing. After collecting explants/tissue samples for DNA extraction, the remaining parts of the specimens were fixed in 95% ethanol and brought to Academia Sinica Museum of Natural History (sample IDs ASIZM0001713, ASIZM0001714, ASIZM0001715, ASIZM0001718, ASIZM0001719). Each specimen was carefully removed from their aquarium tanks and placed on roughly 20 x 20 aluminum foil pieces. We used sterilized scissors or razor blades to dissect four 25 mg muscle tissues from each specimen of either the subepithelial tissue, muscular foot or heart.

Morphological description of gastropods

Aplysia is a genus of sea slugs under the order Anaspidea and family Aplysiidae. Sea slug species have highly reduced internal shells and the body of several *Aplysia* species is characterized by ring-like spots over the head and parapodia. Given this, species identification can be challenging if done morphologically, due to the presence of color and marking polymorphism [2]. In particular, *A. argus* resembles *A. oculifera* and *A. dactylomela*, although the latter is not regarded as occurring in the Indo-Pacific region anymore and the mentioned species can be recognized by their internal shell morphology [2].

Cellana is a limpet (superfamily Patelloida) genus belonging to the family Nacellidae, known for having a singular flattened shell and for being grazers which feed on algae as food [3,4]. *Cellana* is widespread in the Indo-Pacific area [5] and its species were usually recognized by shell morphology, although its high variability made it complex and now usually radular and body characteristics are used instead [4]. As a proof of the complexity of the characters used for species delimitation in this genus, *Ce. orientalis* has a very controversial taxonomy and was once regarded as a subspecies of *Ce. radiata* [6]. The latter is regarded as a species complex [4]. As a

possible morphological marker, Powell [7] reports "very distinct radial folds that underlie the normal radial sculpture" in *Ce. orientalis* (although it was still regarded as a subspecies of *Ce. radiata* at that time). Regarding *Ce. toreuma*, it is widely distributed in East Asia. It presents wide variation in sculptural and color characters, although it presents a depressed conical shape and the aperture is usually oval. It has been present in Asia at least since the Middle Pleistocene [8].

The genus *Conus* is a predatory clade, with more than 750 described species [9]. It has thick coiled shell with the whorls enrolled upon themselves and short shell spire. The aperture is narrow and elongated with parallel margins and they also have a needle-like modified radula with a venom gland to attack and paralyze the prey and then engulf it [9–11]. Both the species we sampled feed on polychaetes [10]. From what concerns *Co. ebraeus*, it superficially resembles *Co. judaeus* due to the presence of black botches on the white shell, although their radula and their feeding preferences (*Co. ebraeus* feeds on Eunicidae, while *Co. judaeus* on syllids) are different [12]*. Co. miles*, instead, share some very vague similarity with *Co. capitaneus* but it has brownish lines around the whorls and spire [10].

Tylothais aculeata is a murex (family Muricidae) snail, which preys on other intertidal invertebrates [13,14]. The shell is coiled and has shell spines [14]. It was recently erected as a standalone genus, due to the fact the previous genus (*Thalessa*) is now regarded as a junior synonym of *Volema* [14]. It was regarded as a *Mancinella* species in Taiwan faunal checklist [15].

Design of student bioinformatic class

Prior to the analysis class, a questionnaire was given to the students to assess their experiences in genome skimming and familiarity with Linux. As approximately half of students had limited or no experiences with Linux, the whole exercise was designed to run under three hours and was conducted by pairs of students with at least one student having some experiences with Linux. The whole exercise is available at [16].

As MitoZ required more time to run, the finished results were prerun and available to students who have completed all the exercise for further interpretation of the annotated results.

Supplementary Figures

Supplementary Figure S1 – Sampling site of gastropods. Gastropods were collected from a rocky shore at Da Bai Sha, Green Island, Taiwan (22.639° N, 121.493° S) and brought back to the Green Island Marine Research Station for further processing.

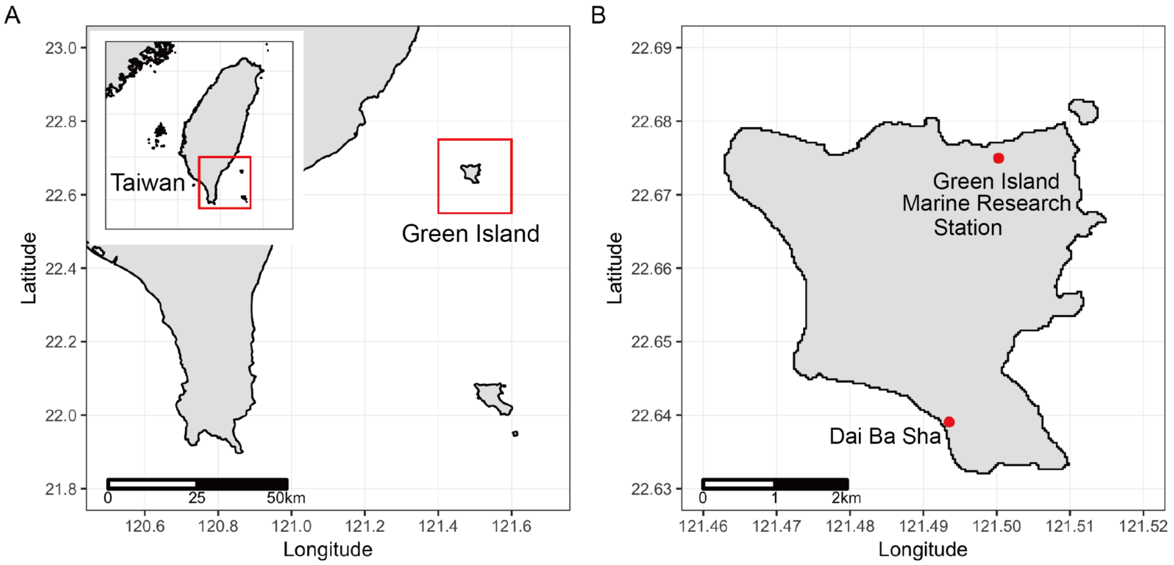

Supplementary Figure S2 – Composition of single-base INDELs in homopolymers.

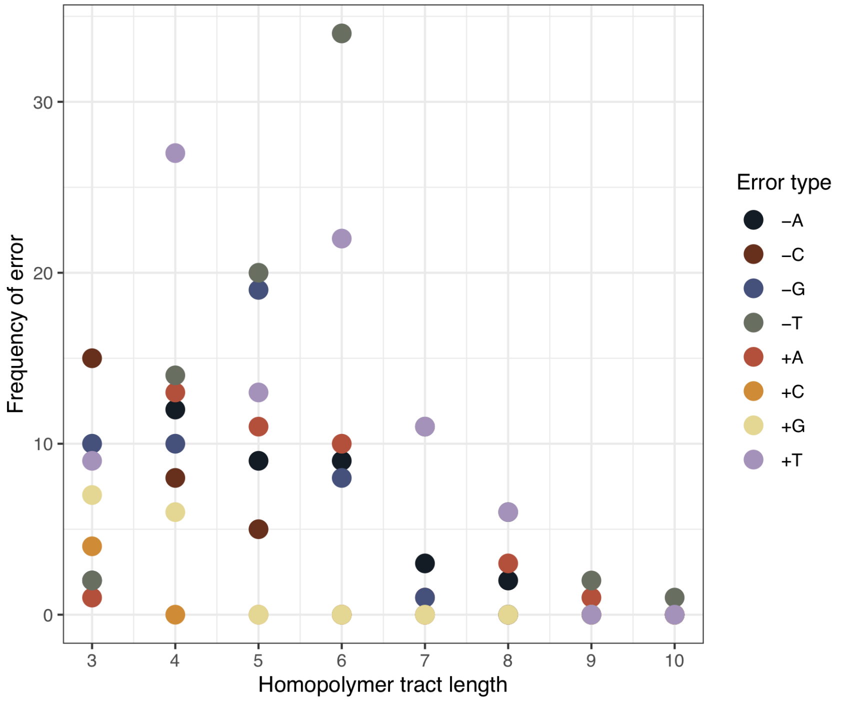

Supplementary Figure S3 – Quantification of ONT errors from hac mode basecalling (A) Number of INDELs (+/-) and substitutions (*) in ONT assemblies before and after consensus improvement using Illumina reads. Error types that occurred once (n=15) and twice (n=8) were excluded from the plot. (B) Relationship between composition of single-base INDELs and homopolymer length.

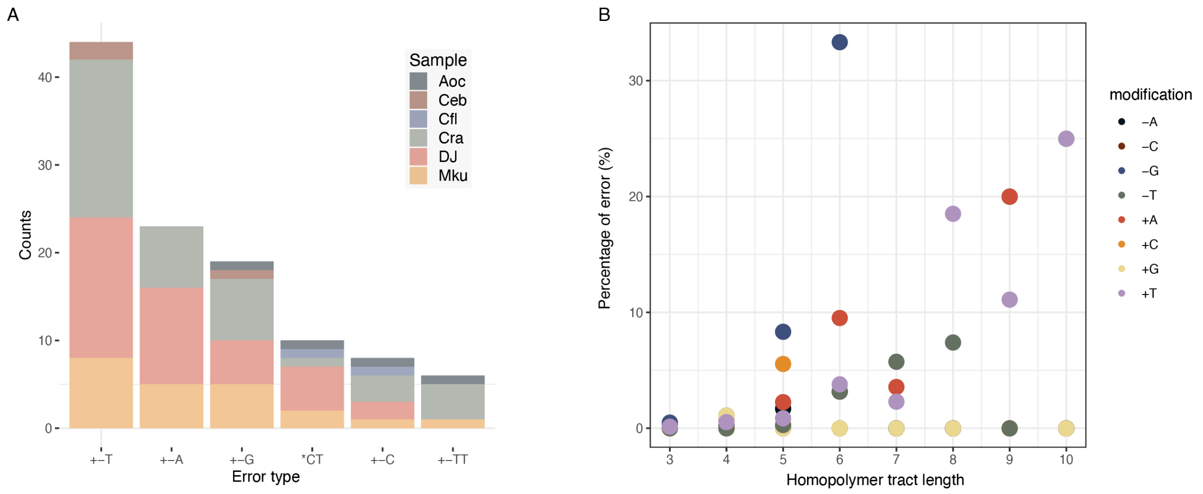

Supplementary Figure S4 – ONT assembly feature of sample Ceb. (A) Dotplot against Illumina assembly. (B) AT content in 50 bp widows. (C) Nanopore and Illumina read coverage in 50 bp windows.

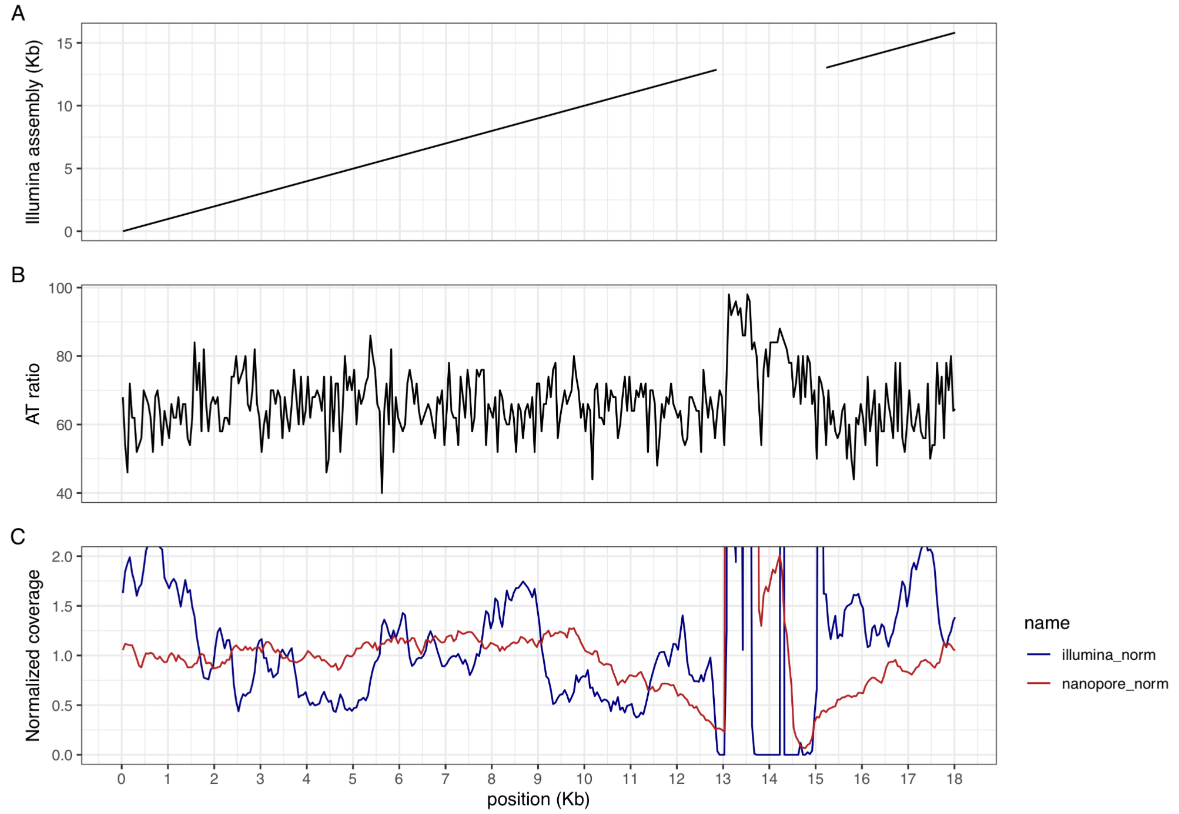

Supplementary Figure S5 – Aplysiidae phylogeny. The topology of the phylogeny was inferred using a coalescence of 13 mitogenome gene phylogenies from ASTRAL [17]. Red colour denotes species sequenced in this study.

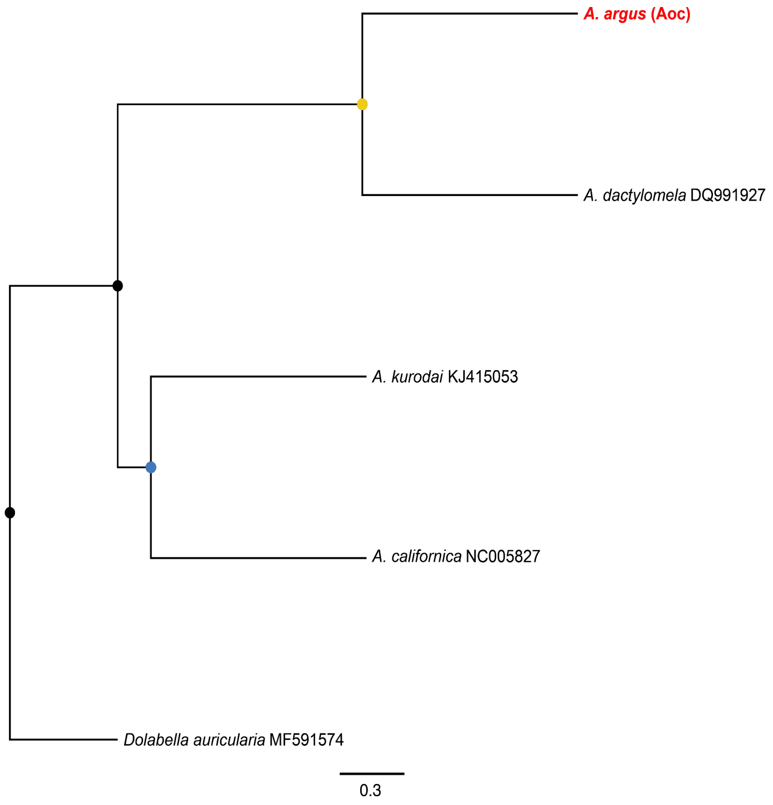

Supplementary Figure S6 – Patellogastropoda phylogeny. The topology of the phylogeny was inferred using a coalescence of 13 mitogenome gene phylogenies from ASTRAL [17]. Red colour denotes species sequenced in this study.

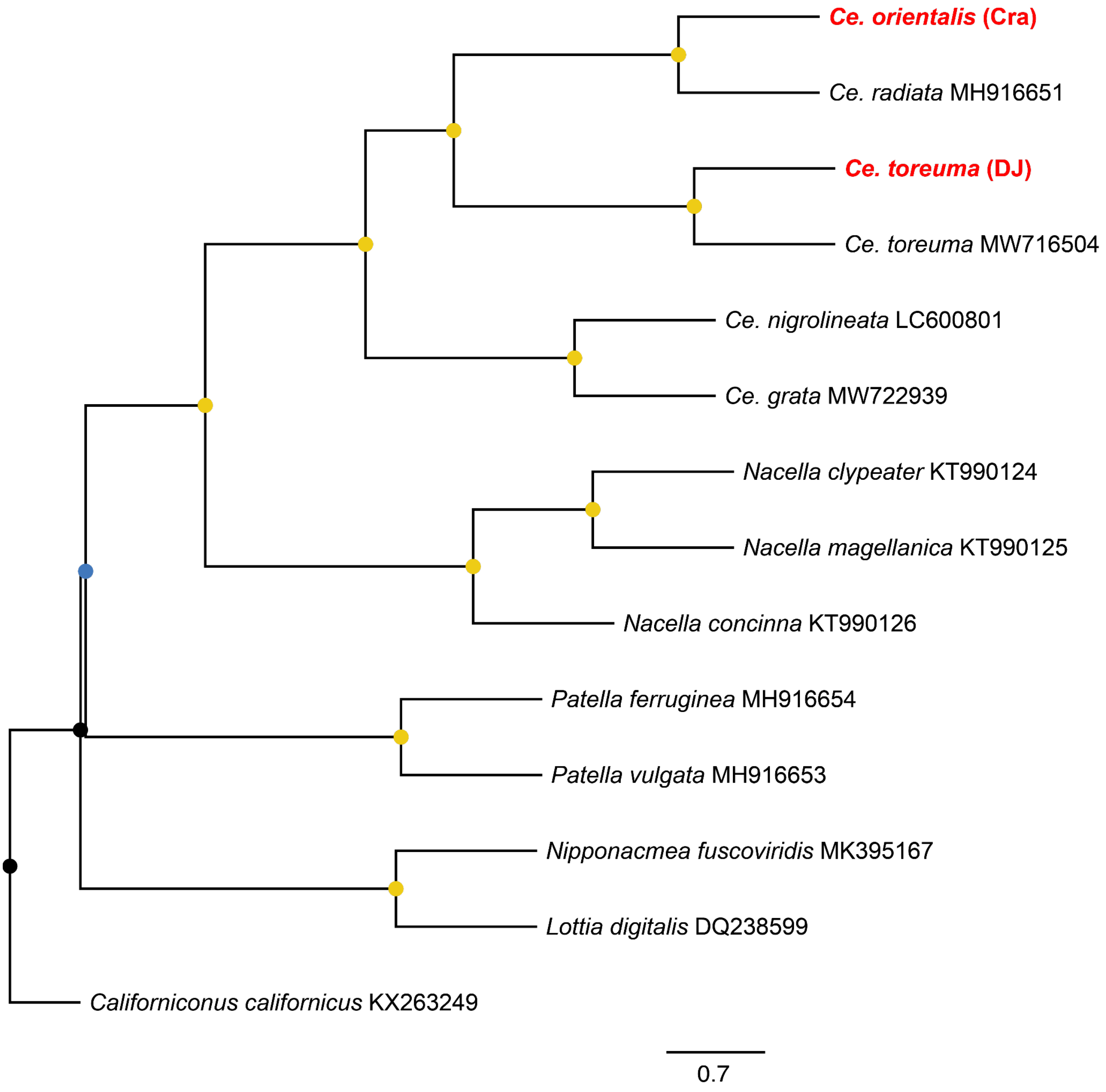

Supplementary Figure S7 – Conidae phylogeny. The topology of the phylogeny was inferred using a coalescence of 13 mitogenome gene phylogenies from ASTRAL [17]. Red colour denotes species sequenced in this study.

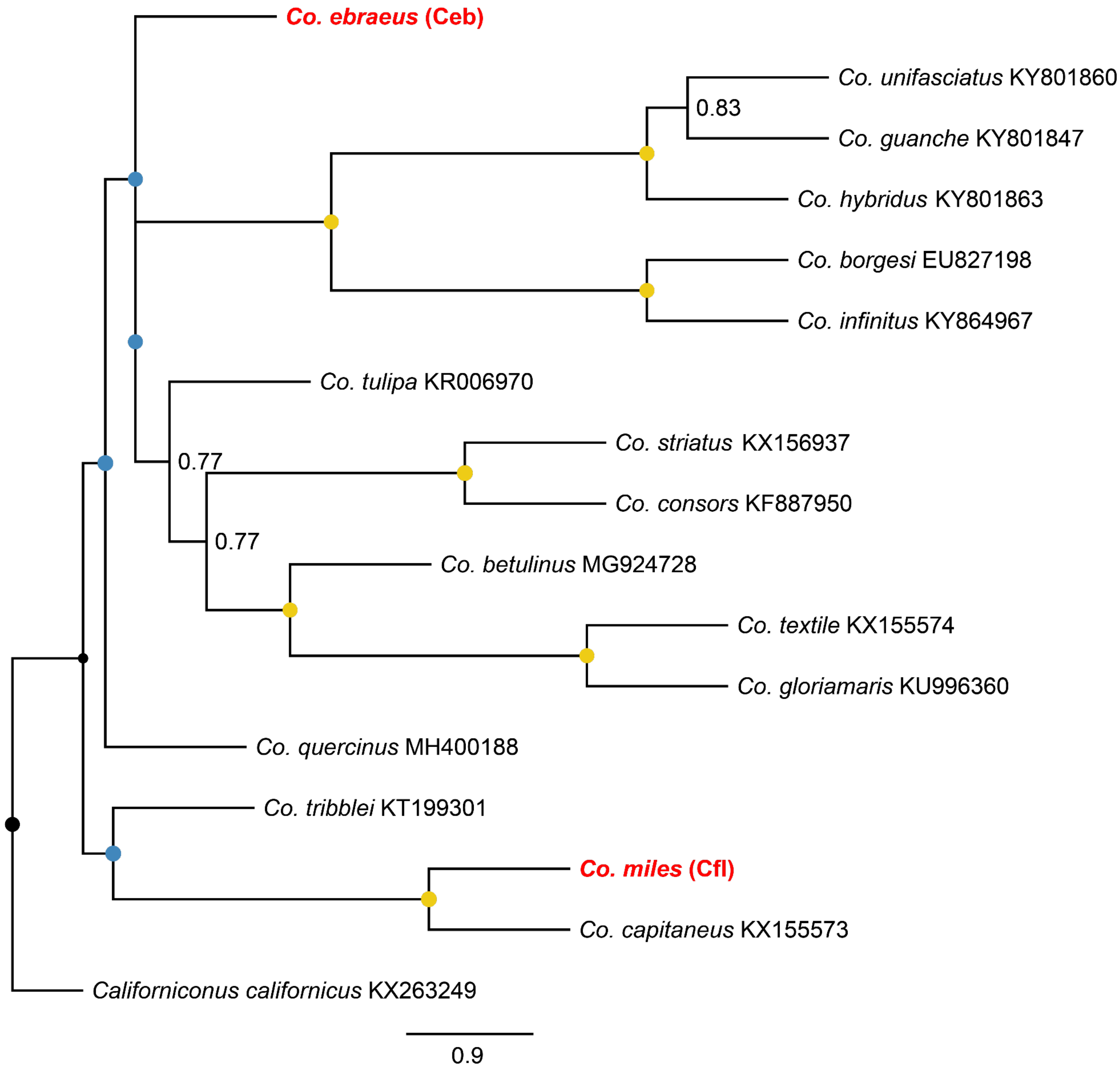

Supplementary Figure S8 – Muricidae phylogeny. The topology of the phylogeny was inferred using a coalescence of 13 mitogenome gene phylogenies from ASTRAL [17]. Red colour denotes species sequenced in this study.

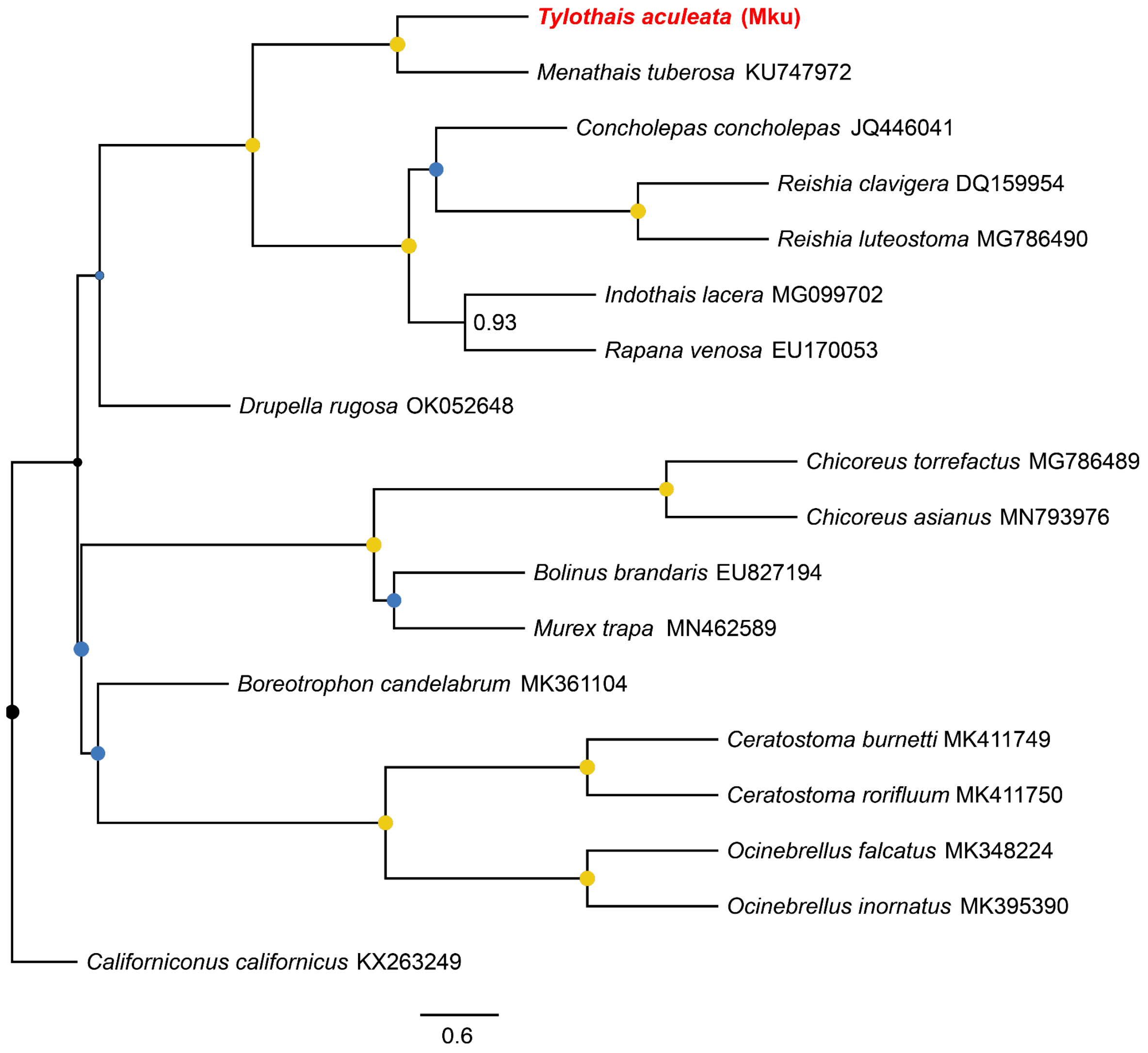

Supplementary Figure S9 – Synteny plot for all the analyzed mitogenomes.

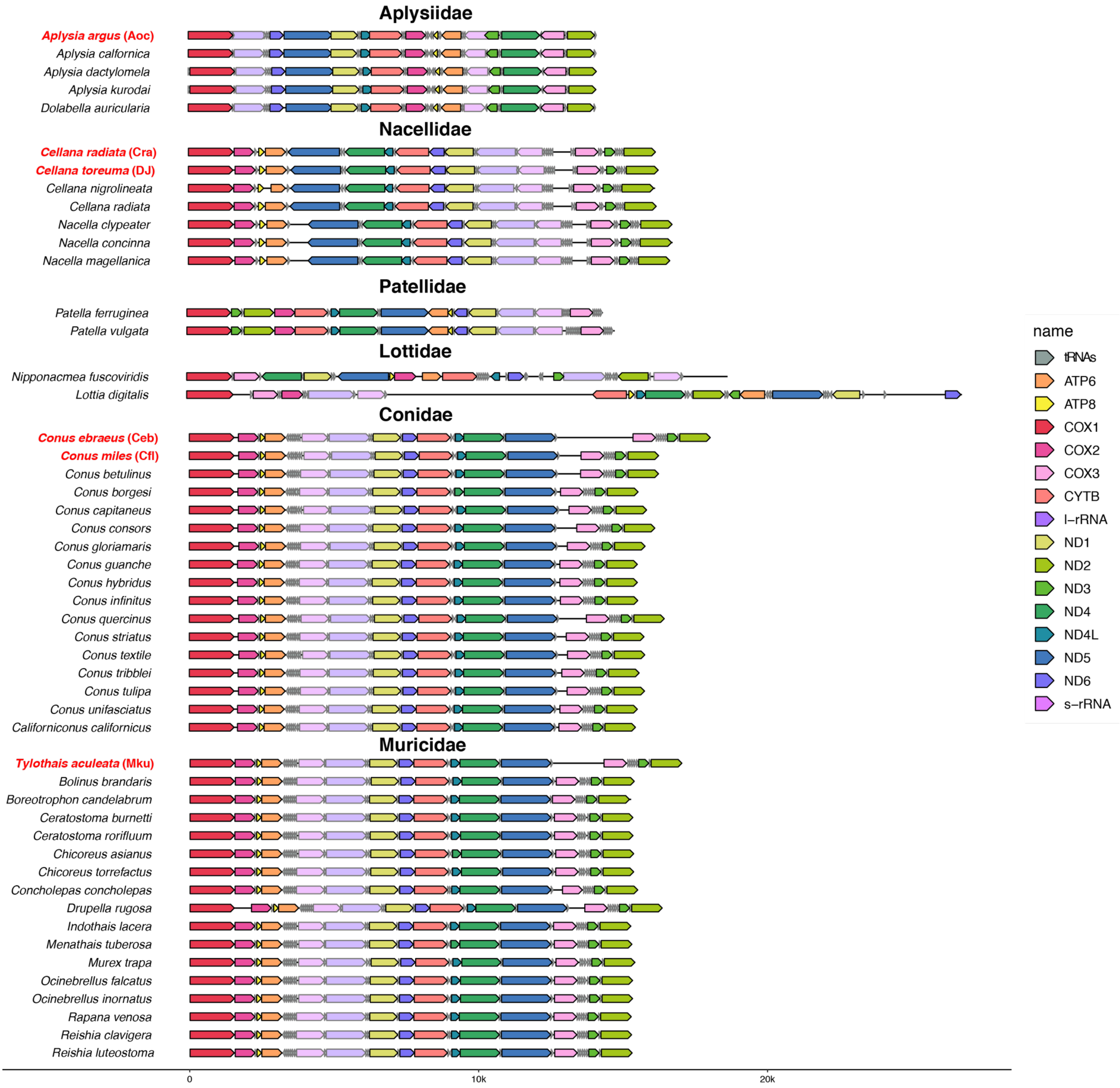

References

1. TIGP-BIODIV. TIGP Signature Course-Ecology Masterclass@Taiwan. https://tigp-biodiv.biodiv.tw/index.php/emt-tigp-signature-course/ (2022).
2. Alexander, J. & Valdés, A. The Ring Doesn't Mean a Thing: Molecular Data Suggest a New Taxonomy for Two Pacific Species of Sea Hares (Mollusca: Opisthobranchia, Aplysiidae). *Pac. Sci.* **67**, 283–294 (2013).
3. Nakano, T. & Ozawa, T. Worldwide phylogeography of limpets of the order Patellogastropoda: molecular, morphological and palaeontological evidence. *J. Molluscan Stud.* **73**, 79–99 (2007).
4. Nakano, T. & Sasaki, T. Recent advances in molecular phylogeny, systematics and evolution of patellogastropod limpets. *J. Molluscan Stud.* **77**, 203–217 (2011).
5. González-Wevar, C. A., Nakano, T., Palma, A. & Poulin, E. Biogeography in *Cellana* (Patellogastropoda, Nacellidae) with special emphasis on the relationships of southern hemisphere oceanic island species. *PLOS ONE* **12**, e0170103; 10.1371/journal.pone.0170103 (2017).
6. WoRMS Editorial Board. World Register of Marine Species. Available from https://www.marinespecies.org at VLIZ. Accessed 2022-01-10. (2022).
7. Powell, A. W. B. The Patellid limpets of the World (Patellidae). in *Indo-Pacific Mollusca* (ed. Abbot, R.) vol. Vol. 3 (The Department of Mollusks, 1973).
8. Kase, T., Nakano, T., Kurihara, Y. & Haga, T. A Middle Pleistocene Limpet Assemblage from Central Japan (Gastropoda: Patellogastropoda) and Selective Extinction of Intertidal Rocky Shore Molluscs in Response to Glacio-Eustatic Sea-Level Changes. *Paleontol. Res.* **17**, 261–281 (2013).
9. Puillandre, N., Duda, T. F., Meyer, C., Olivera, B. M. & Bouchet, P. One, four or 100 genera? A new classification of the cone snails. *J. Molluscan Stud.* **81**, 1–23 (2015).
10. Röckel, D., Korn, W. & Kohn, A. J. *Manual of the Living Conidae*. vol. 1 (Verlag Christa Hemmen, 1995).

11. Uribe, J. E., Puillandre, N. & Zardoya, R. Beyond *Conus*: Phylogenetic relationships of Conidae based on complete mitochondrial genomes. *Mol. Phylogenet. Evol.* **107**, 142–151 (2017).
12. Duda, T. F., Kohn, A. J. & Matheny, A. M. Cryptic Species Differentiated in *Conus ebraeus* , a Widespread Tropical Marine Gastropod. *Biol. Bull.* **217**, 292–305 (2009).
13. Claremont, M., Vermeij, G. J., Williams, S. T. & Reid, D. G. Global phylogeny and new classification of the Rapaninae (Gastropoda: Muricidae), dominant molluscan predators on tropical rocky seashores. *Mol. Phylogenet. Evol.* **66**, 91–102 (2013).
14. Houart, R. Description of eight new species and one new genus of Muricidae (Gastropoda) from the Indo-West Pacific. *Novapex* **18**, 81–103 (2017).
15. Shao, K.-T. & Chung, K.-F. The National Checklist of Taiwan (Catalogue of Life in Taiwan, TaiCoL). *GBIF*. https://www.gbif.org/dataset/1ec61203-14fa-4fbd-8ee5-a4a80257b45a (2021).
16. Tsai, I. J. Genome skimming exercise (last updated 2022.04.14). https://introtogenomics.readthedocs.io/en/latest/emcgs.html. (2022).
17. Rabiee, M., Sayyari, E. & Mirarab, S. Multi-allele species reconstruction using ASTRAL. *Mol. Phylogenet. Evol.* **130**, 286–296 (2019).
